# Variable Transposition of Eight Maize *Activator (Ac*) Elements Located on the Short Arm of Chromosome 1

**DOI:** 10.1534/g3.111.000729

**Published:** 2011-09-01

**Authors:** William F. Sheridan

**Affiliations:** Department of Biology, University of North Dakota, Grand Forks, North Dakota 58202

**Keywords:** maize, *Ac* elements, transposition rate, variability

## Abstract

Eight Activator (*Ac*) transposable elements mapped to the maize chromosome arm 1S were assessed for *Ac* transposition rates. For each of the *Ac* stocks, plants homozygous for the single *Ac* element and the Ds reporter *r1-sc:m3* on chromosome 10 were crossed as females by a homozygous *r1-sc:m3* tester color-converted W22 line. The resulting ears produced mostly coarsely spotted kernels and a low frequency of either near-colorless fine-spotted kernels or nonspotted kernels. The relative frequency of these two types of near-colorless kernels differed among the eight *Ac* stocks. The extent to which increased *Ac* dosage results in nonspotted kernels may be *Ac*-specific. Although all of the *Ac* elements are in near-isogenic inbred W22 lines, they varied to a large extent in their transposition frequency. These differences might possibly result from structural differences among the *Ac* elements. Because one pair of *Ac* elements derived from *Ac33* on chromosome arm 5S differed about 13-fold in transposition frequency and a second pair of *Ac* elements derived from Ac12 on chromosome arm 1S differed about 3-fold in transposition frequency, this is not a likely explanation for all eight *Ac* elements. The data presented here support the notion that the differences in transposition frequency of the eight *Ac* elements may be a reflection of variability in *Ac* transcription or accessibility of the transposase to the *Ac* element, resulting from differences in the chromatin environments wherein the *Ac* elements are located. This is the first report of variability in transposition rates among different *Ac* donor lines.

Transposon tagging with the maize *Ac* element is a useful tool for regional mutagenesis (Federoff *et al.* 1984; Brutnell and Conrad 2003; Singh *et al.* 2003). Two features of the *Ac* element make it a tractable system for gene tagging. There are several genes controlling anthocyanin synthesis in the aluerone and embryo that contain *Ds* element insertions and can serve as reporter loci for the presence of an *Ac* element (McClintock 1955; Dooner *et al.* 1994). In addition, the delayed timing of *Ac* transposition in tissues containing increased *Ac* copy number provides a means of assessing the occurrence of an *Ac* transposition event by observing the size of pigmented sectors in the aleurone and embryo tissues of kernels.

## MATERIALS AND METHODS

**Figure 1:**
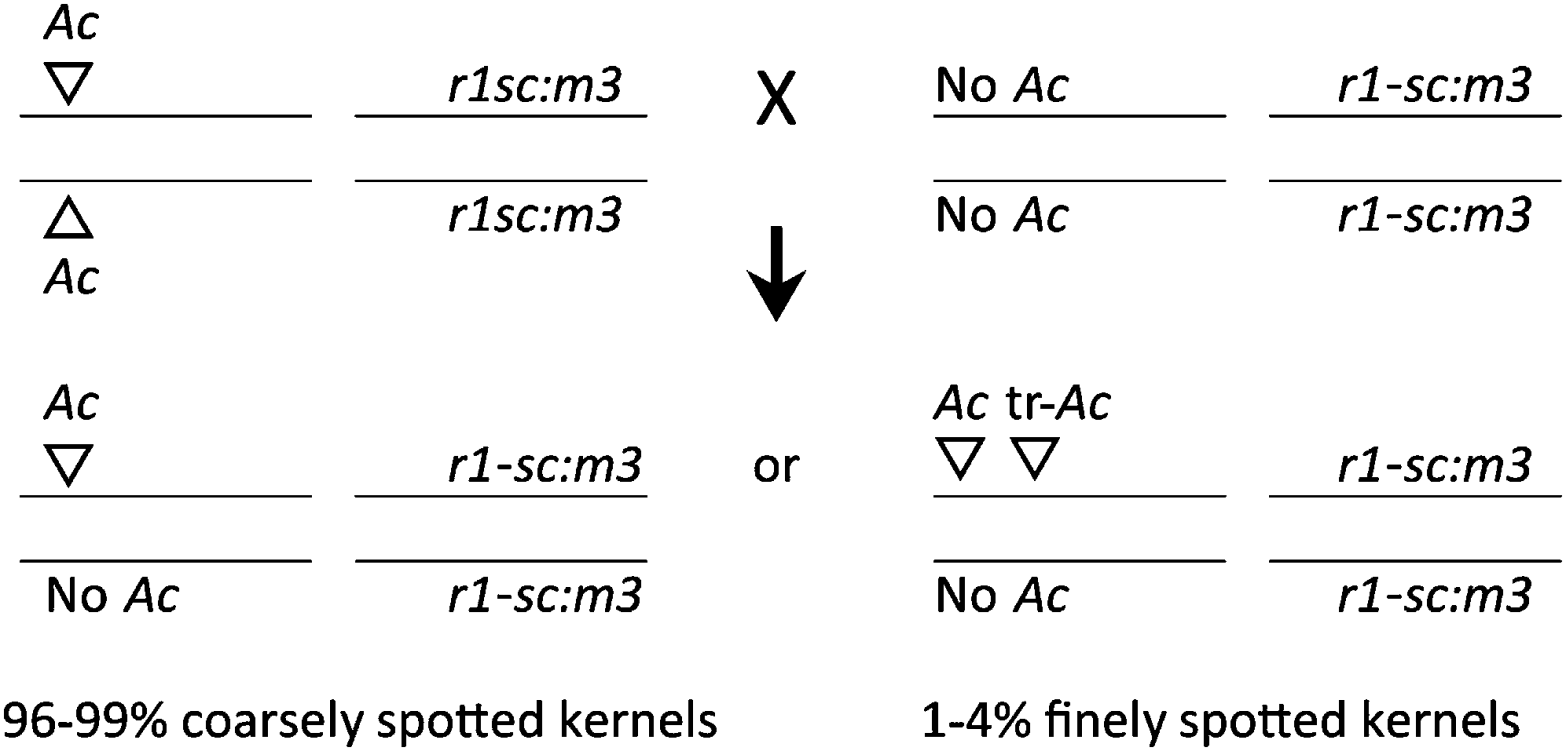
Crossing scheme to produce finely spotted kernels. The chromosome constitutions of the embryos are shown. In most cases, the *Ac* element does not transpose and the embryo contains a single copy of the *Ac* element, whereas the aleurone cells of the endosperm contain two copies (one *Ac* from each of the two polar nuclei of the embryo sac). This dosage results in coarse spotting of the kernel aleurones. In a few cases, the *Ac* element duplicates, and subsequently, one copy transposes to a new site. When both copies are transmitted to the embryo sac (whether they are linked as shown in the figure or are on different chromosomes), the embryo will have two copies of the *Ac* element and the aleurone will have four copies, resulting in a finely spotted aleurone.

A collection of *Ac*-containing, near-isogenic, color-converted W22 inbred lines was produced by Kolkman *et al.* (2005); these included 41 precisely mapped *Ac* elements. The present report concerns 8 of these lines, all containing *Ac* elements mapped to the short arm of chromosome 1. The results presented here concern the variability in transposition frequency of these *Ac* elements observed while pursuing a regional mutagenesis program on this chromosome arm. For each of the *Ac* stocks, plants homozygous for the single *Ac* element and the *Ds* reporter *r1-sc:m3* on chromosome 10 were crossed as females by a homozygous *r1-sc:m3* tester color-converted W22 line (Figure 1).

### Scoring of ears for *Ac* transposition events

**Figure 2:**
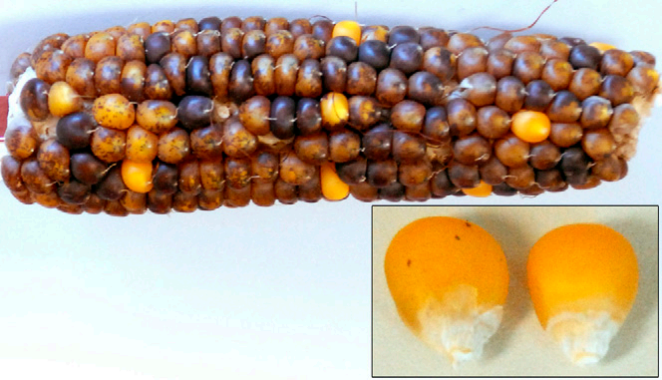
Results of crossing an *Ac* stock as shown in Figure 1 and generating transposed *Ac*s. An F1 ear produced by crossing a stock homozygous for *mon00068::Ac* by the *r1-sc:m3* reporter tester line. Most of the kernels have an aleurone layer containing two copies of the *Ac* element and are coarsely spotted. A small number of kernels have an aleurone layer containing four copies of the *Ac* element (two copies of the original *Ac* element and two copies of the newly transposed *Ac* element) and are finely spotted (near-colorless).

The ears borne on plants homozygous for the *Ac* element produce mostly coarsely spotted kernels and a low frequency of near-colorless kernels. The coarsely spotted kernels display the pattern of colored sectors expected when the nuclei of the female gametophyte contain a single copy of the *Ac* element, whereas the near-colorless kernels manifest either a transposition event resulting in an increased dosage of the *Ac* element in these nuclei or the absence of an *Ac* element. Increased dosage may result from the *Ac* element replicating prior to its transposition and the subsequent presence of both the donor *Ac* element and the *tr-Ac* element in the functional megaspore that develops into the female gametophyte (embryo sac) (Greenblatt 1968). The increased dosage of the *Ac* element results in the delay in the development of the fine spotting of colored sectors in these kernels (see Figure 2). A total of 394 scoreable ears were screened for near-colorless kernels. Each such kernel was scored as a transposition event and was removed from the ear and examined for fine spots under magnification. The kernels were sorted into two groups: spotted kernels containing at least one fine spot on either the aleurone or the embryo, and nonspotted kernels lacking any colored spots on both aleurone and the embryo (Figure 2).

Initially, the transposition frequencies were calculated on the basis of transposition events per ear. The number of ears scored for each of the *Ac* stocks ranged from 24 to 77. Among the eight *Ac* stocks, the mean values ranged from a high frequency of 9.67 fine-spotted kernels and 6.34 nonspotted kernels per ear (*bti00252::Ac*) to a low frequency of 0.58 fine-spotted kernels and 0.03 nonspotted kernels per ear (*mon00106::Ac*) (supporting information, Table S1, Figure S1). Subsequently, all of the kernels on the scored ears were weighed to provide an estimate of the total number of kernels per ear. The total estimated number of kernels examined was approximately 108,000. These data were used to calculate an estimated frequency of fine-spotted and nonspotted kernels per 1000 kernels for each of the eight *Ac* elements. The mean values ranged from a high frequency of 38.80 fine-spotted kernels and 26.28 nonspotted kernels per 1000 kernels (*bti00252::Ac*) to a low frequency of 2.15 fine-spotted kernels and 0.15 nonspotted kernels per 1000 kernels (*mon00106::Ac*) (Table 1). The frequency of transposition of *Ac* elements per 1000 kernels for the individual families of the eight *Ac* elements is shown in Table S2.

**Table 1:** Frequency of transposition of *Ac* elements per 1000 kernels from eight sites on maize chromosome arm 1S

Donor *Ac* element	Source of *Ac**^a^*	Bin location	Number of ears scored	Total number of kernels scored	Number of fine spotted kernels per 1000 kernels	Number of nonspotted kernels per 1000 kernels
mon03080	*r-nj:m1* 10L	1.02	31	9411	11.66 ± 2.3 bc*^b^*	24.35 ± 2.96 ac
bti95004	*P1-vv* 1S	1.02/0.03	51	13774	13.57 ± 1.48 bc	20.85 ± 1.79 abc
mon00106	*Ac33* 5S	1.02/0.03	77	21257	2.15 ± 0.37 e	0.15 ± 0.15 f
bti00228	*P1-vv* 1S	1.03	71	19032	9.67 ± 0.97 b	7.43 ± 1.26 d
mon00192	*Ac12* 1S	1.03	58	16629	12.74 ± 1.17 bc	10.71 ± 1.25 de
bti95006	*P1-vv* 1S	1.03	25	6470	18.85 ± 1.96 c	15.22 ± 2.75 cde
bti00252	*Ac12* 1S	1.04/0.05	24	6121	38.80 ± 3.84 a	26.28 ± 3.28 a
mon00068	*Ac33* 5S	1.05	57	15100	27.81 ± 1.96 d	16.49 ± 1.69 be

^a^ The source of the *Ac* elements that transposed into the mapped sites on chromosome arm 1S are presented together with the chromosome arm location of the source *Ac* element (Kevin Ahern, personal communication).

^b^ Mean values with the same letter are not significantly different at the 0.05 level. See Table S3 and Table S4 for ANOVA and Tukey comparisons.

### Variation in *Ac* dosage effects

An average transposition frequency of 2 to 4% (20 to 40 per 1000 kernels) was reported by Kolkman *et al.* (2005) based on their examination of approximately 12,400 kernels generated from 10 different *Ac* lines. No data were provided for any of the individual lines. In this report, the *Ac* transposition frequency as evidenced by the frequency of near-colorless, fine-spotted kernels ranged from 0.215% to 3.880%. When the near-colorless nonspotted kernels are included in the calculations, then the frequency of *Ac* transposition ranges from 0.230% to 6.508%. Kolkman *et al.* (2005) noted differences in the degree of variation in *Ac*-mediated *Ds* variegation patterns (the size of sectors or spots) in kernels homozygous for independent *Ac* insertions. These researchers further noted that, inasmuch as all of the *Ac* elements they studied were in near-isogenic lines using the same *Ds* reporter, it was not likely that the variations they observed resulted from differences in the reporter gene or segregating modifier loci.

## RESULTS

The size of spots on self-pollinated kernels on ears of plants homozygous for the eight different *Ac* stocks differed only slightly in size, and there was no obvious relationship with their *Ac* transposition frequency. The same degree of similarity of spotting size was observed on kernels on ears of plants homozygous for *Ac* elements that were crossed as females by the reporter stock. When plants homozygous for *Ac* elements were crossed by pollen of the reporter stock, the relative frequency of fine-spotted to nonspotted kernels differed among the eight *Ac* elements (Table 1, Figure S1). In the case of the two most distally located *Ac* elements (*mon03080::Ac* and *bti95004::Ac*), the number of nonspotted kernels per 1000 kernels was greater than the number of spotted kernels per 1000 kernels. However, for the six most proximally located *Ac* elements, the frequency of spotted kernels was greater than the frequency of nonspotted kernels. The relative frequency of nonspotted kernels may depend on the level of transposase transcription by the *Ac* elements, and this level may vary among the *Ac* elements at both their original sites on chromosome arm 1S and at the target sites of the *tr-Ac* elements. Where the sum of the resulting transposase levels is high enough, then the negative dosage effect (Heinlein 1996; Kunze and Weil 2002) exerted upon transposition of *Ds* from the reporter locus may suppress all spotting, even though *Ac* elements are present in the kernel. However, inasmuch as the differences in spotting frequency are a reflection of the frequency of the transposition of the *Ds* element from the *r1-sc:m3* reporter locus, these differences may not be directly linked to the frequency of transposition of the *Ac* element. Whereas the accessibility of the transposase to the *Ds* element is likely to be similar in these isogenic lines, the accessibility of the *Ac* element to transposase may differ among the *Ac* elements and may be influenced by its position in the chromosome. Consequently, the extent to which an increased *Ac* dosage results in nonspotted kernels may be *Ac* element–specific but not directly related to *Ac* transposition frequency.

The same considerations regarding the use of isogenic lines containing the same *Ds* reporter apply to the present study wherein significant differences in *Ac* transposition frequencies are documented (Table 1, Table S3, Table S4). It was earlier noted by Kolkman *et al.* (2005) that previous studies have indicated that methylation plays an important role in altering transcription patterns of *Ac* and that, therefore, the variation in *Ac*-mediated excision patterns of the *Ds* element is likely a result of differences in the transcriptional activity of the different *Ac* elements and that their mapped *Ac* lines may be useful for analyzing “*cis*-acting elements that control gene expression throughout the genome.” The differences in *Ac* transposition frequency reported here might possibly result from structural differences among the *Ac* elements. However, this is not a likely explanation for all eight cases. An examination of Table 1 reveals that the source of both *mon00106::Ac* and *mon00068::Ac* was *Ac33* on chromosome arm 5S, yet their transposition frequencies differed about 13-fold. Likewise, the source of both *mon00192::Ac* and *bti00252::Ac* was *Ac12* on chromosome arm 1S, yet their transposition frequencies differed about 3-fold. The data presented here support the notion that the differences in transposition frequency of these eight mapped *Ac* elements may be a reflection of variability in *Ac* transcription or accessibility of transposase to the *Ac* element, resulting from differences in the chromatin environments wherein the *Ac* elements are located.

## Supplementary Material

Supporting Information
