# Supplementary material for: Variable Transposition of Eight Maize Activator (Ac) Elements Located on the Short Arm of Chromosome 1
Source: G3 (Bethesda). 2011 Sep 1;1(4):259–61. doi: 10.1534/g3.111.000729 (PMC3276147; doi:10.1534/g3.111.000729)
Supplement: Supporting Information [file supp_1_4_259__index.html]

Supporting Information 

# Variable Transposition of Eight Maize *Activator (Ac*) Elements Located on the Short Arm of Chromosome 1

## Supporting Information for Sheridan, 2011

**Files in this Data Supplement:**

- Supporting Information - Figure S1 and Tables S1-S4 (PDF, 372 KB)
- Figure S1 - The mean number of spotted and nonspotted kernels per scored ear (PDF, 128 KB)
- Table S1 - Frequency of transposition of *Ac* elements per ear from eight sites on maize chromosome arm 1S (PDF, 44 KB)
- Table S2 - Frequency of transposition of *Ac* elements per 1000 kernels for the individual families of the eight *Ac* elements (PDF, 68 KB)
- Table S3 - Tukey's comparisons for fine spotted kernels (PDF, 76 KB)
- Table S4 - Tukey's comparisons for nonspotted kernels (PDF, 68 KB)
