# Supplementary material for: Variable Transposition of Eight Maize Activator (Ac) Elements Located on the Short Arm of Chromosome 1
Source: G3 (Bethesda). 2011 Sep 1;1(4):259–61. doi: 10.1534/g3.111.000729 (PMC3276147; doi:10.1534/g3.111.000729)
Supplement: Supporting Information [file supp_1.4.259_000729SI.pdf]

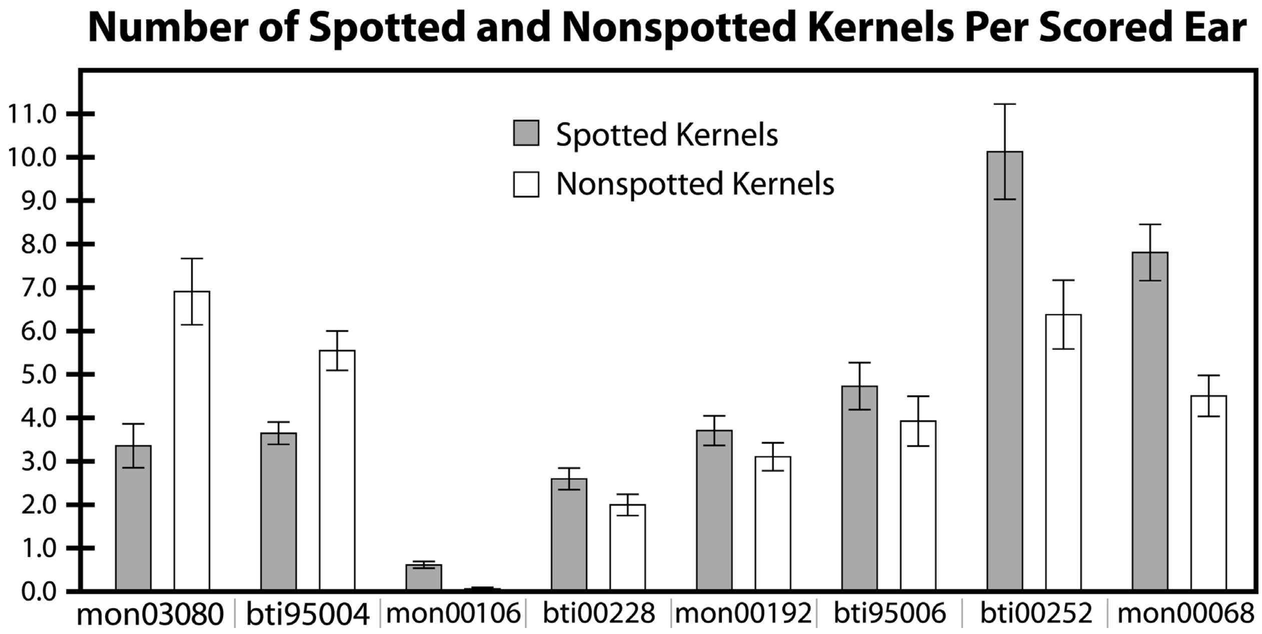

Figure S1 The mean number of spotted and nonspotted kernels per scored ear. Standard error of the mean is indicated for all values. Two pairs of elements that originated from the same progenitor *Ac* element but have large differences in transposition frequencies are *mon00106::Ac* and *mon00068::Ac* (from *Ac33* on 5S) and *mon00192::Ac* and *bti00252::Ac* (from *Ac12* on1S). These differences may result from differences in the chromatin features flanking them.

Table S1   Frequency of transposition of *Ac* elements per ear from eight sites on maize chromosome arm 1S

Ac stocks*	Bin Location	Number of scored ears	Number of near-colorless kernels	Number of fine spotted kernels	Mean number of fine spotted kernels per ear	Number of nonspotted kernels	Mean number of nonspotted kernels per ear
mon03080	1.02	31	316	103	3.32±0.56	213	6.87*±0.79
bti95004	1.02/.03	51	465	184	3.61±0.31	281	5.51±0.47
mon00106	1.02/.03	77	47	45	0.58±0.09	2	0.03±0.02
bti00228	1.03	71	321	182	2.59±0.26	139	1.93±0.26
mon00192	1.03	58	398	221	3.81±0.38	177	3.05±0.31
bti95006	1.03	25	220	121	4.84±0.57	99	3.96±0.63
bti00252	1.04/.05	24	378	232	9.67±1.09	146	6.34±0.88
mon00068	1.05	57	698	443	7.72±.07	255	4.47±0.49
**Total**		**394**	**2843**	**1531**		**1312**	
Mean		49.25	7.22	3.89		3.33	

*The SEM value is shown as ± values next to each mean value

Table S2 Frequency of transposition of *Ac* elements per 1000 kernels for the individual families of the eight *Ac* elements

Ac element	Family No.	Number of ears scored	Number of kernels scored	Number of fine spotted kernels per 1000 kernels	Number of nonspotted kernels per 1000 kernels
mon03080	97	17	4626	16.27±3.82	35.24±3.50
	98	14	4785	6.07±1.43	11.13±1.42
	97+98	31	9411	11.66±2.35	24.35±2.96
	t-test P-values*			0.028	1.993E-06
bti95004	99	20	5458	12.08±1.86	21.15±2.66
	100	31	8316	14.52±1.46	20.65±2.42
	99+100	51	13774	13.57±1.15	20.85±1.79
	t-test P-values			0.303	0.894
mon00106	101	19	4304	2.74±0.68	0
	102	10	3036	2.88±1.73	1.163±1.16
	103	48	13917	1.76±0.41	0
	101+102+103	77	21257	2.15±0.37	0.15±0.15
	Anova P-values			0.410	0.033
bti00228	104	35	9605	10.15±1.44	7.30±1.22
	105	36	9427	9.20±1.33	7.55±2.20
	104+105	71	19032	9.67±0.97	7.43±1.26
	t-test P-values			0.629	0.923
mon00192	106	54	15564	12.32±1.20	10.37±1.28
	107	4	1065	18.38±5.00	15.36±5.70
	106+107	58	16629	12.74±1.17	10.71±1.25
	t-test P-values			0.192	0.317
bti95006	108	9	2833	24.63±3.55	23.48±4.04
	110	16	3637	15.59±1.97	10.57±3.16
bti00252	108+110	25	6470	18.85±1.96	15.22±2.75
	t-test P-values			0.023	0.021
	112	13	3252	41.77±3.52	30.12±4.72
	113	7	1776	48.83±7.45	24.27±4.57
	115	4	1093	11.59±2.99	17.33±9.35
	112+113+115	24	6121	38.80±3.84	26.28±3.28
	Anova P-values			0.001	0.367
mon0068	116	17	4347	26.57±4.38	15.08±3.03
	117	15	4218	33.07±3.75	17.70±3.95
	118	25	6535	20.80±3.20	17.89±3.41

116+117+118	57	15100	27.81±1.96	16.49±1.69
Anova P-values			0.274	0.843

*Tests for heterogeneity among the families of each of the *Ac* elements were performed using the students t-test (two families) and Anova analysis (three families).

TABLE S3 Tukey's comparisons for fine spotted kernels

Ac element	*Ac* element	Estimate	Std. Error	t value	Pr(>|t|)
bti00252	bti00228	29.1248	2.4420	11.926	< 0.001 ***
bti95004	bti00228	3.8931	1.8984	2.051	0.43939
bti95006	bti00228	9.1751	2.4053	3.815	0.00370 **
mon00068	bti00228	18.1384	1.8393	9.861	< 0.001 ***
mon00106	bti00228	-7.5244	1.7017	-4.422	< 0.001 ***
mon00192	bti00228	3.0637	1.8305	1.674	0.69610
mon03080	bti00228	1.9920	2.2265	0.895	0.98573
bti95004	bti00252	-25.2317	2.5602	-9.856	< 0.001 ***
bti95006	bti00252	-19.9497	2.9556	-6.750	< 0.001 ***
mon00068	bti00252	-10.9864	2.5167	-4.365	< 0.001 ***
mon00106	bti00252	-36.6492	2.4179	-15.158	< 0.001 ***
mon00192	bti00252	-26.0611	2.5102	-10.382	< 0.001 ***
mon03080	bti00252	-27.1328	2.8120	-9.649	< 0.001 ***
bti95006	bti95004	5.2820	2.5251	2.092	0.41268
mon00068	bti95004	14.2453	1.9935	7.146	< 0.001 ***
mon00106	bti95004	-11.4175	1.8672	-6.115	< 0.001 ***
mon00192	bti95004	-0.8294	1.9854	-0.418	0.99989
mon03080	bti95004	-1.9011	2.3554	-0.807	0.99228
mon00068	bti95006	8.9634	2.4810	3.613	0.00773 **
mon00106	bti95006	-16.6995	2.3807	-7.014	< 0.001 ***
mon00192	bti95006	-6.1114	2.4745	-2.470	0.20458
mon03080	bti95006	-7.1830	2.7802	-2.584	0.15915
mon00106	mon00068	-25.6628	1.8072	-14.201	< 0.001 ***
mon00192	mon00068	-15.0747	1.9290	-7.815	< 0.001 ***
mon03080	mon00068	-16.1464	2.3081	6.996	< 0.001 ***
mon00192	mon00106	10.5881	1.7982	5.888	< 0.001 ***
mon03080	mon00106	9.5165	2.1999	4.326	< 0.001 ***
mon03080	mon00192	-1.0717	2.3010	-0.466	0.99977

Significance codes: 0 '***' 0.001 '**' 0.01 '*' 0.05 '.' 0.1 ' ' 1

(Adjusted p values reported -- single-step methods)

Significance grouping for *Ac* elements- fine spotted kernels

bti00252	bti95004	bti95006	mon00068	mon00106	mon00192	mon03080	bti00228
"a"	"bc"	"c"	"d"	"e"	"bc"	"bc"	"b"

(Significance is at the $p \leq 0.05$ level)

Anova. Comparison of means for fine spotted kernels

	Df	Sum Sq	Mean Sq	F value	Pr(>F)
Ac Elements	7	38600	5514.4	51.552	< 2.2e-16***
Residuals	386	41289	107.0		

Table S4 Tukey's comparisons for nonspotted kernels

Ac element	*Ac* element	Estimate	Std. Error	t value	Pr(>|t|)
bti00252	bti00228	18.855	2.662	7.083	< 0.001 ***
bti95004	bti00228	13.420	2.069	6.485	< 0.001 ***
bti95006	bti00228	7.789	2.622	2.971	0.05958 .
mon00068	bti00228	9.059	2.005	4.518	< 0.001 ***
mon00106	bti00228	-7.276	1.855	-3.923	0.00261 **
mon00192	bti00228	3.286	1.995	1.647	0.71303
mon03080	bti00228	16.927	2.427	6.975	< 0.001 ***
bti95004	bti00252	-5.434	2.791	-1.947	0.50942
bti95006	bti00252	-11.066	3.222	-3.435	0.01419 *
mon00068	bti00252	-9.796	2.743	-3.571	0.00914 **
mon00106	bti00252	-26.130	2.636	-9.915	< 0.001 ***
mon00192	bti00252	-15.568	2.736	-5.690	< 0.001 ***
mon03080	bti00252	-1.928	3.065	-0.629	0.99838
bti95006	bti95004	-5.632	2.752	-2.046	0.44195
mon00068	bti95004	-4.361	2.173	-2.007	0.46843
mon00106	bti95004	-20.696	2.035	-10.169	< 0.001 ***
mon00192	bti95004	-10.134	2.164	-4.683	< 0.001 ***
mon03080	bti95004	3.507	2.567	1.366	0.86729
mon00068	bti95006	1.270	2.704	0.470	0.99976
mon00106	bti95006	-15.064	2.595	-5.805	< 0.001 ***
mon00192	bti95006	-4.502	2.697	-1.669	0.69880
mon03080	bti95006	9.138	3.030	3.016	0.05246 .
mon00106	mon00068	-16.335	1.970	-8.293	< 0.001 ***
mon00192	mon00068	-5.773	2.103	-2.746	0.10818
mon03080	mon00068	7.868	2.516	3.127	0.03769 *
mon00192	mon00106	10.562	1.960	5.389	< 0.001 ***
mon03080	mon00106	24.203	2.398	10.093	< 0.001 ***
mon03080	mon00192	13.641	2.508	5.439	< 0.001 ***

Significance codes: 0 '***' 0.001 '**' 0.01 '*' 0.05 '.' 0.1 ' ' 1

(Adjusted p values reported -- single-step methods)

Significance Grouping for Ac elements- nonspotted kernels

bti00252	bti95004	bti95006	mon00068	mon00106	mon00192	mon03080	bti00228
"a"	"abc"	"cde"	"be"	"f"	"de"	"ac"	"d"

(Significance is at the $p \leq 0.05$ level)

Anova. Comparison of means for nonspotted kernels

	Df	Sum Sq	Mean Sq	F value	Pr(>F)
Ac Elements	7	27312	3901.7	30.701	< 2.2e-16 ***
Residuals	386	49056	127.1		
