# Supplementary material for: Variable Transposition of Eight Maize Activator (Ac) Elements Located on the Short Arm of Chromosome 1
Source: G3 (Bethesda). 2011 Sep 1;1(4):259–61. doi: 10.1534/g3.111.000729 (PMC3276147; doi:10.1534/g3.111.000729)
Supplement: Supporting Information [file supp_1.4.259_FigureS1.pdf]

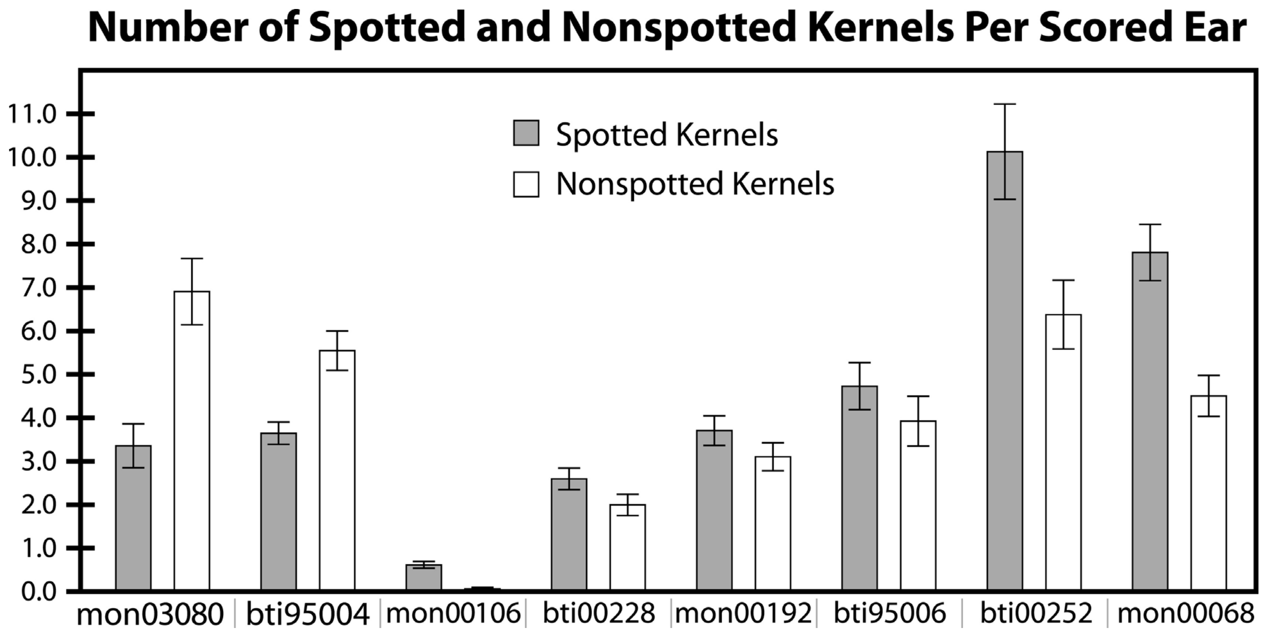

Figure S1 The mean number of spotted and nonspotted kernels per scored ear. Standard error of the mean is indicated for all values. Two pairs of elements that originated from the same progenitor *Ac* element but have large differences in transposition frequencies are *mon00106::Ac* and *mon00068::Ac* (from *Ac33* on 5S) and *mon00192::Ac* and *bti00252::Ac* (from *Ac12* on1S). These differences may result from differences in the chromatin features flanking them.
