# Supplementary material for: Variable Transposition of Eight Maize Activator (Ac) Elements Located on the Short Arm of Chromosome 1
Source: G3 (Bethesda). 2011 Sep 1;1(4):259–61. doi: 10.1534/g3.111.000729 (PMC3276147; doi:10.1534/g3.111.000729)
Supplement: Supporting Information [file supp_1.4.259_TableS1.pdf]

**Table S1   Frequency of transposition of *Ac* elements per ear from eight sites on maize chromosome arm 1S**

| *Ac* stocks* | Bin Location | Number of scored ears | Number of near-colorless kernels | Number of fine spotted kernels | Mean number of fine spotted kernels per ear | Number of nonspotted kernels | Mean number of nonspotted kernels per ear |
|---|---|---|---|---|---|---|---|
| mon03080 | 1.02 | 31 | 316 | 103 | 3.32±0.56 | 213 | 6.87*±0.79 |
| bti95004 | 1.02/.03 | 51 | 465 | 184 | 3.61±0.31 | 281 | 5.51±0.47 |
| mon00106 | 1.02/.03 | 77 | 47 | 45 | 0.58±0.09 | 2 | 0.03±0.02 |
| bti00228 | 1.03 | 71 | 321 | 182 | 2.59±0.26 | 139 | 1.93±0.26 |
| mon00192 | 1.03 | 58 | 398 | 221 | 3.81±0.38 | 177 | 3.05±0.31 |
| bti95006 | 1.03 | 25 | 220 | 121 | 4.84±0.57 | 99 | 3.96±0.63 |
| bti00252 | 1.04/.05 | 24 | 378 | 232 | 9.67±1.09 | 146 | 6.34±0.88 |
| mon00068 | 1.05 | 57 | 698 | 443 | 7.72±.07 | 255 | 4.47±0.49 |
| **Total** | | **394** | **2843** | **1531** | | **1312** | |
| Mean | | 49.25 | 7.22 | 3.89 | | 3.33 | |

*The SEM value is shown as ± values next to each mean value
