# Supplementary material for: Variable Transposition of Eight Maize Activator (Ac) Elements Located on the Short Arm of Chromosome 1
Source: G3 (Bethesda). 2011 Sep 1;1(4):259–61. doi: 10.1534/g3.111.000729 (PMC3276147; doi:10.1534/g3.111.000729)
Supplement: Supporting Information [file supp_1.4.259_TableS2.pdf]

Table S2 Frequency of transposition of *Ac* elements per 1000 kernels for the individual families of the eight *Ac* elements

Ac element	Family No.	Number of ears scored	Number of kernels scored	Number of fine spotted kernels per 1000 kernels	Number of nonspotted kernels per 1000 kernels
mon03080	97	17	4626	16.27±3.82	35.24±3.50
	98	14	4785	6.07±1.43	11.13±1.42
	97+98	31	9411	11.66±2.35	24.35±2.96
	t-test P-values*			0.028	1.993E-06
bti95004	99	20	5458	12.08±1.86	21.15±2.66
	100	31	8316	14.52±1.46	20.65±2.42
	99+100	51	13774	13.57±1.15	20.85±1.79
	t-test P-values			0.303	0.894
mon00106	101	19	4304	2.74±0.68	0
	102	10	3036	2.88±1.73	1.163±1.16
	103	48	13917	1.76±0.41	0
	101+102+103	77	21257	2.15±0.37	0.15±0.15
	Anova P-values			0.410	0.033
bti00228	104	35	9605	10.15±1.44	7.30±1.22
	105	36	9427	9.20±1.33	7.55±2.20
	104+105	71	19032	9.67±0.97	7.43±1.26
	t-test P-values			0.629	0.923
mon00192	106	54	15564	12.32±1.20	10.37±1.28
	107	4	1065	18.38±5.00	15.36±5.70
	106+107	58	16629	12.74±1.17	10.71±1.25
	t-test P-values			0.192	0.317
bti95006	108	9	2833	24.63±3.55	23.48±4.04
	110	16	3637	15.59±1.97	10.57±3.16
bti00252	108+110	25	6470	18.85±1.96	15.22±2.75
	t-test P-values			0.023	0.021
	112	13	3252	41.77±3.52	30.12±4.72
	113	7	1776	48.83±7.45	24.27±4.57
	115	4	1093	11.59±2.99	17.33±9.35
	112+113+115	24	6121	38.80±3.84	26.28±3.28
	Anova P-values			0.001	0.367
mon0068	116	17	4347	26.57±4.38	15.08±3.03
	117	15	4218	33.07±3.75	17.70±3.95
	118	25	6535	20.80±3.20	17.89±3.41

	116+117+118	57	15100	27.81±1.96	16.49±1.69
	Anova P-values			0.274	0.843

*Tests for heterogeneity among the families of each of the *Ac* elements were performed using the students t-test (two families) and Anova analysis (three families).
