# Supplementary material for: Variable Transposition of Eight Maize Activator (Ac) Elements Located on the Short Arm of Chromosome 1
Source: G3 (Bethesda). 2011 Sep 1;1(4):259–61. doi: 10.1534/g3.111.000729 (PMC3276147; doi:10.1534/g3.111.000729)
Supplement: Supporting Information [file supp_1.4.259_TableS3.pdf]

TABLE S3 Tukey's comparisons for fine spotted kernels

Ac element	*Ac* element	Estimate	Std. Error	t value	Pr(>|t|)
bti00252	bti00228	29.1248	2.4420	11.926	< 0.001 ***
bti95004	bti00228	3.8931	1.8984	2.051	0.43939
bti95006	bti00228	9.1751	2.4053	3.815	0.00370 **
mon00068	bti00228	18.1384	1.8393	9.861	< 0.001 ***
mon00106	bti00228	-7.5244	1.7017	-4.422	< 0.001 ***
mon00192	bti00228	3.0637	1.8305	1.674	0.69610
mon03080	bti00228	1.9920	2.2265	0.895	0.98573
bti95004	bti00252	-25.2317	2.5602	-9.856	< 0.001 ***
bti95006	bti00252	-19.9497	2.9556	-6.750	< 0.001 ***
mon00068	bti00252	-10.9864	2.5167	-4.365	< 0.001 ***
mon00106	bti00252	-36.6492	2.4179	-15.158	< 0.001 ***
mon00192	bti00252	-26.0611	2.5102	-10.382	< 0.001 ***
mon03080	bti00252	-27.1328	2.8120	-9.649	< 0.001 ***
bti95006	bti95004	5.2820	2.5251	2.092	0.41268
mon00068	bti95004	14.2453	1.9935	7.146	< 0.001 ***
mon00106	bti95004	-11.4175	1.8672	-6.115	< 0.001 ***
mon00192	bti95004	-0.8294	1.9854	-0.418	0.99989
mon03080	bti95004	-1.9011	2.3554	-0.807	0.99228
mon00068	bti95006	8.9634	2.4810	3.613	0.00773 **
mon00106	bti95006	-16.6995	2.3807	-7.014	< 0.001 ***
mon00192	bti95006	-6.1114	2.4745	-2.470	0.20458
mon03080	bti95006	-7.1830	2.7802	-2.584	0.15915
mon00106	mon00068	-25.6628	1.8072	-14.201	< 0.001 ***
mon00192	mon00068	-15.0747	1.9290	-7.815	< 0.001 ***
mon03080	mon00068	-16.1464	2.3081	6.996	< 0.001 ***
mon00192	mon00106	10.5881	1.7982	5.888	< 0.001 ***
mon03080	mon00106	9.5165	2.1999	4.326	< 0.001 ***
mon03080	mon00192	-1.0717	2.3010	-0.466	0.99977

Significance codes: 0 '***' 0.001 '**' 0.01 '*' 0.05 '.' 0.1 ' ' 1

(Adjusted p values reported -- single-step methods)

Significance grouping for *Ac* elements- fine spotted kernels

bti00252	bti95004	bti95006	mon00068	mon00106	mon00192	mon03080	bti00228
"a"	"bc"	"c"	"d"	"e"	"bc"	"bc"	"b"

(Significance is at the $p \leq 0.05$ level)

Anova. Comparison of means for fine spotted kernels

	Df	Sum Sq	Mean Sq	F value	Pr(>F)
Ac Elements	7	38600	5514.4	51.552	< 2.2e-16***
Residuals	386	41289	107.0		
