# Supplementary material for: Variable Transposition of Eight Maize Activator (Ac) Elements Located on the Short Arm of Chromosome 1
Source: G3 (Bethesda). 2011 Sep 1;1(4):259–61. doi: 10.1534/g3.111.000729 (PMC3276147; doi:10.1534/g3.111.000729)
Supplement: Supporting Information [file supp_1.4.259_TableS4.pdf]

**Table S4 Tukey's comparisons for nonspotted kernels**

| *Ac* element | *Ac* element | Estimate | Std. Error | t value | Pr(>\|t\|) |
|---|---|---|---|---|---|
| bti00252 | bti00228 | 18.855 | 2.662 | 7.083 | < 0.001 *** |
| bti95004 | bti00228 | 13.420 | 2.069 | 6.485 | < 0.001 *** |
| bti95006 | bti00228 | 7.789 | 2.622 | 2.971 | 0.05958 . |
| mon00068 | bti00228 | 9.059 | 2.005 | 4.518 | < 0.001 *** |
| mon00106 | bti00228 | -7.276 | 1.855 | -3.923 | 0.00261 ** |
| mon00192 | bti00228 | 3.286 | 1.995 | 1.647 | 0.71303 |
| mon03080 | bti00228 | 16.927 | 2.427 | 6.975 | < 0.001 *** |
| bti95004 | bti00252 | -5.434 | 2.791 | -1.947 | 0.50942 |
| bti95006 | bti00252 | -11.066 | 3.222 | -3.435 | 0.01419 * |
| mon00068 | bti00252 | -9.796 | 2.743 | -3.571 | 0.00914 ** |
| mon00106 | bti00252 | -26.130 | 2.636 | -9.915 | < 0.001 *** |
| mon00192 | bti00252 | -15.568 | 2.736 | -5.690 | < 0.001 *** |
| mon03080 | bti00252 | -1.928 | 3.065 | -0.629 | 0.99838 |
| bti95006 | bti95004 | -5.632 | 2.752 | -2.046 | 0.44195 |
| mon00068 | bti95004 | -4.361 | 2.173 | -2.007 | 0.46843 |
| mon00106 | bti95004 | -20.696 | 2.035 | -10.169 | < 0.001 *** |
| mon00192 | bti95004 | -10.134 | 2.164 | -4.683 | < 0.001 *** |
| mon03080 | bti95004 | 3.507 | 2.567 | 1.366 | 0.86729 |
| mon00068 | bti95006 | 1.270 | 2.704 | 0.470 | 0.99976 |
| mon00106 | bti95006 | -15.064 | 2.595 | -5.805 | < 0.001 *** |
| mon00192 | bti95006 | -4.502 | 2.697 | -1.669 | 0.69880 |
| mon03080 | bti95006 | 9.138 | 3.030 | 3.016 | 0.05246 . |
| mon00106 | mon00068 | -16.335 | 1.970 | -8.293 | < 0.001 *** |
| mon00192 | mon00068 | -5.773 | 2.103 | -2.746 | 0.10818 |
| mon03080 | mon00068 | 7.868 | 2.516 | 3.127 | 0.03769 * |
| mon00192 | mon00106 | 10.562 | 1.960 | 5.389 | < 0.001 *** |
| mon03080 | mon00106 | 24.203 | 2.398 | 10.093 | < 0.001 *** |
| mon03080 | mon00192 | 13.641 | 2.508 | 5.439 | < 0.001 *** |

Significance codes: 0 '***' 0.001 '**' 0.01 '*' 0.05 '.' 0.1 ' ' 1

(Adjusted p values reported -- single-step methods)

Significance Grouping for Ac elements- nonspotted kernels

| bti00252 | bti95004 | bti95006 | mon00068 | mon00106 | mon00192 | mon03080 | bti00228 |
|---|---|---|---|---|---|---|---|
| "a" | "abc" | "cde" | "be" | "f" | "de" | "ac" | "d" |

(Significance is at the $p \leq 0.05$ level)

Anova. Comparison of means for nonspotted kernels

| | Df | Sum Sq | Mean Sq | F value | Pr(>F) |
|---|---|---|---|---|---|
| Ac Elements | 7 | 27312 | 3901.7 | 30.701 | < 2.2e-16 *** |
| Residuals | 386 | 49056 | 127.1 | | |
